# Relationship of Soluble Interleukin-6 Receptors With Asthma: A Mendelian Randomization Study

**DOI:** 10.3389/fmed.2021.665057

**Published:** 2021-04-12

**Authors:** Yoshihiko Raita, Zhaozhong Zhu, Carlos A. Camargo, Robert J. Freishtat, Debby Ngo, Liming Liang, Kohei Hasegawa

**Affiliations:** ^1^Department of Emergency Medicine, Massachusetts General Hospital and Harvard Medical School, Boston, MA, United States; ^2^Division of Emergency Medicine, Children's National Hospital, Washington, DC, United States; ^3^Department of Pediatrics, George Washington University School of Medicine and Health Sciences, Washington, DC, United States; ^4^Department of Genomics and Precision Medicine, George Washington University School of Medicine and Health Sciences, Washington, DC, United States; ^5^Pulmonary, Critical Care and Sleep Medicine, Beth Israel Deaconess Medical Center, Boston, MA, United States; ^6^Program in Genetic Epidemiology and Statistical Genetics, Department of Epidemiology, Harvard T.H. Chan School of Public Health, Boston, MA, United States; ^7^Department of Biostatistics, Harvard T.H. Chan School of Public Health, Boston, MA, United States

**Keywords:** interleukin-6, soluble interleukin-6 receptor, trans-signaling pathway, asthma, Mendelian randomization, GWAS, UK Biobank

## Abstract

**Purpose:** Emerging evidence suggests a potential role of interleukin-6 pathways—trans-signaling with soluble interleukin-6 receptors—in the asthma pathobiology. Despite the evidence for their associations with asthma, the causal role of soluble interleukin-6 receptors remains uncertain. We investigated the relations of soluble interleukin-6 receptors with asthma and its major phenotypes.

**Methods:** We conducted a two-sample Mendelian randomization study. As genetic instruments, we selected 33 independent *cis*-acting variants strongly associated with the level of plasma soluble interleukin-6 receptor in the INTERVAL study. To investigate the association of variants with asthma and its phenotypes, we used genome-wide association study data from the UK Biobank. We combined variant-specific causal estimates by the inverse-variance weighted method for each outcome.

**Results:** Genetically-instrumented soluble interleukin-6 receptor level was associated with a significantly higher risk of overall asthma (OR per one standard deviation increment in inverse-rank normalized soluble interleukin-6 receptor level, 1.02; 95%CI, 1.01–1.03; *P* = 0.004). Sensitivity analyses demonstrated consistent results and indicated no directional pleiotropy—e.g., MR-Egger (OR, 1.03; 95%CI, 1.01–1.05; *P* = 0.002; *P*_intercept_ =0.37). In the stratified analysis, the significant association persisted across asthma phenotypes—e.g., childhood asthma (OR, 1.05; 95%CI, 1.02–1.08; *P* < 0.001) and obese asthma (OR, 1.02; 95%CI 1.01–1.03; *P* = 0.007). Sensitivity analysis using 16 variants selected with different thresholds also demonstrated significant associations with overall asthma and its phenotypes.

**Conclusion:** Genetically-instrumented soluble interleukin-6 receptor level was causally associated with modestly but significantly higher risks of asthma and its phenotypes. Our observations support further investigations into identifying specific endotypes in which interleukin-6 pathways may play major roles.

## Introduction

Among many immune components involved in the pathobiology of asthma, recent research has suggested a potential role of interleukin-6 (IL-6) signaling—the classic and trans-signaling pathways (1). The trans-signaling pathway starts with coupling with IL-6 and soluble IL-6 receptor (sIL-6R), and formation of a complex with the ubiquitously expressed membrane-bound glycoprotein 130, thereby activating downstream pro-inflammatory cascades—e.g., Janus kinase-signal transducer and activator of transcription (JAK-STAT) pathway (1). This IL-6 trans-signaling pathway plays major roles in a range of inflammatory conditions (e.g., rheumatic diseases, inflammatory bowel diseases, obesity), and is the target of anti-IL-6 therapies (e.g., tocilizumab) (1).

Epidemiologic studies have found associations of an increased IL-6 level in the serum, sputum, and bronchoalveolar lavage fluid with asthma prevalence and its severity (2–4). Genome-wide association studies (GWAS) have also reported that the single nucleotide polymorphism (SNP) rs2228145 (Asp^358^Ala)—a variant in *IL6R* that increases IL-6R shedding and promotes IL-6 trans-signaling—is associated with asthma prevalence, asthma severity, and lower pulmonary function (5, 6). Moreover, rs4129267—which has a perfect linkage disequilibrium with rs2228145 above in European subjects—is also known as an asthma susceptibility locus (7). Despite the evidence on these *associations* which may suffer from unmeasured confounding, the *causal* role of sIL-6R in asthma (and hence the potential role of anti-IL-6R therapies) remains uncertain. To address the knowledge gap in the literature, we performed an instrumental variable analysis with genetic instruments (i.e., Mendelian randomization) to examine the effect of sIL-6R on asthma and its major phenotypes.

## Methods

This is a two-sample Mendelian randomization study using GWAS summary statistics from two large cohort studies—the INTERVAL study (8) and UK Biobank (9–12). Detailed Methods can be found in the Supplementary Material. In brief, Mendelian randomization can provide unbiased causal estimates in an observation study because the genetic polymorphisms associated with the exposure (sIL-6R levels) are allocated randomly at conception and its causal inference is less susceptible to confounding and reverse causation (13).

## Data Summary

### The Interval Study

The INTERVAL study is a prospective cohort study that recruited approximately 50,000 blood donors aged ≥18 years. For the proteomic profiling, randomly selected two non-overlapping sub-cohorts of 2,731 and 831 participants of European ancestry were enrolled. The levels of 2,994 plasma proteins were measured by the use of SomaLogic assays. A genome-wide protein quantitative trait loci (pQTL) analysis of 2,994 plasma proteins in 3,301 healthy adults of European ancestry was conducted (8). Overall, 1,927 significant (P_GWAS_ <1.5 × 10^−11^) associations between 764 genomic regions and 1,478 proteins were identified. The summary statistics data are publicly available at http://www.phpc.cam.ac.uk/ceu/proteins/.

### The UK Biobank

The UK Biobank is a prospective cohort study that enrolled approximately 500,000 adults (aged 40–69 years at enrollment in 2006–2010), and collected comprehensive phenotypic data and performed genome-wide genotyping (9). The current analysis restricted the sample to 394,256 subjects of European ancestry to minimize population stratification (46,799 cases with asthma and 347,457 controls). In the current study, the primary outcome was (overall) asthma (*n* = 46,799). The secondary outcomes were six major asthma phenotypes: (1) childhood asthma (defined as age of onset ≤ 12 years; *n* = 9,676) (12), (2) adult-onset asthma (defined as age of onset ≥26 years; *n* = 22,294) (12), (3) allergic asthma (defined as asthma with an allergic disease—eczema, food allergy, and/or allergy rhinitis [identified by data fields 6152, 20002, 41202, 41204]; *n* = 23,183) (10, 11), (4) non-allergic asthma (defined as asthma without any allergic disease; *n* = 23,616), (5) obese asthma (defined as BMI of ≥30 kg/m^2^; *n* = 13,550), and (6) non-obese asthma (defined as BMI of <30 kg/m^2^; *n* = 33,095). We also identified shared controls (*n* = 347,457) with high-quality genotyping and complete phenotype and covariate data for GWAS association analysis. All participants from this study provided UK Biobank-acquired informed consent and provided data according to the UK Biobank protocol. The institutional review board of Harvard University and Massachusetts General Hospital approved the study.

### Statistical Analysis

As the genetic instruments, we identified 33 independent *cis*-acting variants strongly associated with plasma sIL-6R levels (P_GWAS_ <5 × 10^−6^, *r*^2^ <0.1, 250kb from *IL6R*; Supplementary Table 1) in the INTERVAL study (mean age, 44 years; female, 49%) (8). All variants had an *F-*statistic of >10, without a significant association with major confounders (such as education status, smoking, and physical activity; Supplementary Table 1) nor a known pleiotropy in Ensembl, GWAS catalog, and PhenoScanner. Separately, using the UK Biobank data, we computed the GWAS statistics for asthma and six major asthma phenotypes, as previously described (10–12).

To investigate the association of variants with outcomes, we used GWAS summary statistics of the UK Biobank. We weighted the magnitude of association of each variant with outcomes by that with sIL-6R, and combined causal estimates of sIL-6R on each outcome by the inverse-variance weighted meta-analysis method with a random-effects model (14, 15) by using *MendelianRandomization* package (16).

In the sensitivity analyses, we first applied MR-Egger regression (17), MR Pleiotropy RESidual Sum and Outlier (MR-PRESSO) test (18), and MR weighted median method (19). MR-Egger regression detects pleiotropy based on the assumption that the pleiotropic associations are independent from the genetic associations with the exposure (i.e., the instrument strength independent of direct effect [InSIDE] assumption) and provides corrected estimates. MR-PRESSO test (18) detects violation of the restriction exclusion criterion assumption and provides corrected estimates by removing variants which exhibit pleiotropy. MR weighted median method provides consistent estimates even when 50% of the information comes from invalid variants. We conducted MR-Egger regression and weighted median method using *MendelianRandomization* package (16) and MR-PRESSO using *MRPRESSO* package (18). Second, we also used more-stringent P_GWAS_ (P_GWAS_ <5 × 10^−8^) and linkage disequilibrium (*r*^2^ <0.02) thresholds to select genetic instruments in order to examine the robustness of the inferences. We analyzed the data using R version 3.6.3 (R foundation for Statistical Computing, Vienna, Austria).

## Results

**Figure 1:**
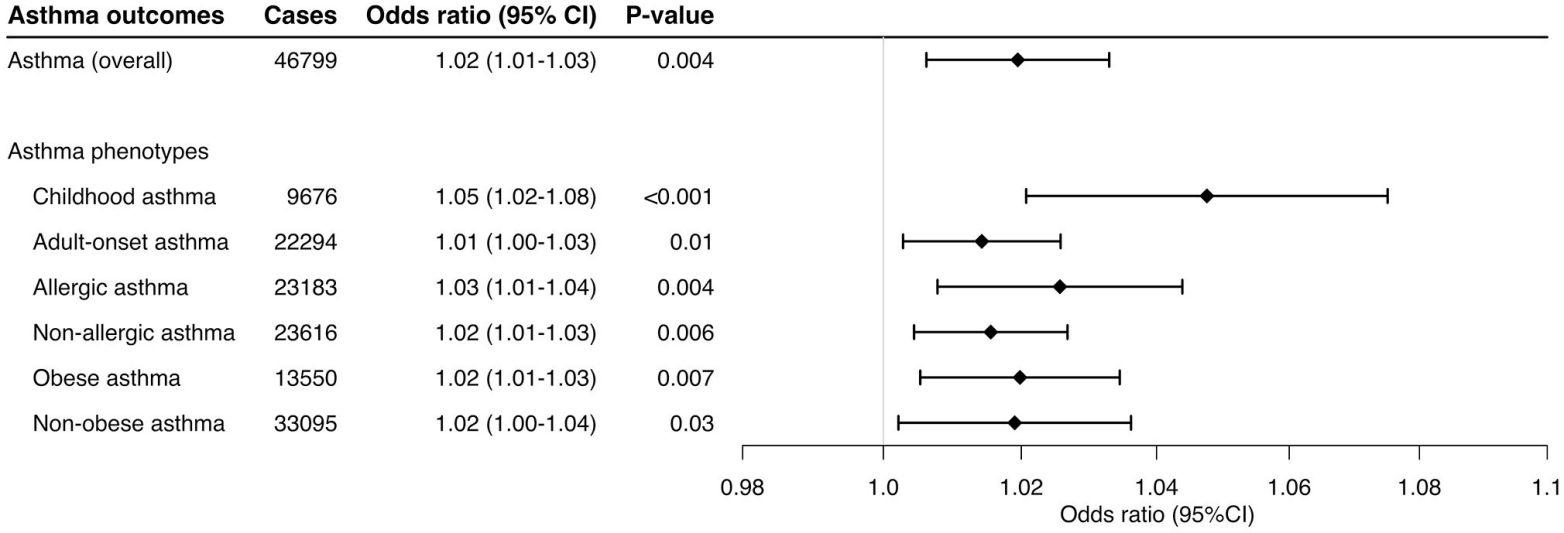
Mendelian randomization estimates for the effect of soluble interleukin-6 receptors on overall asthma outcome. The size of the squares is proportional to the weight of the Mendelian randomization estimate for each variant, with the horizontal lines indicating their 95% confidence intervals. The center of the diamond represents the combined Mendelian randomization point estimate with the lateral tips indicating its 95% confidence interval, estimated by the inverse variance weighted method. The odds ratios were estimated per one standard deviation increment in the inverse-rank normalized sIL-6R level. CI, confidence interval; IVW, inverse variance weighted.

Higher genetically-instrumented sIL-6R levels were associated with a modestly but significantly increased risk of overall asthma (OR per one standard deviation increment in inverse-rank normalized sIL-6R level, 1.02; 95%CI, 1.01–1.03; *P* = 0.004; Figure 1) with the use of inverse-variance weighted meta-analysis method. Of the 33 genetic instruments, rs4129267—an asthma susceptibility locus^7^ with a linkage disequilibrium of *r*^2^=1 with rs2228145 (Asp^358^Ala) (6)—had the largest weight on the Mendelian randomization estimate. The sensitivity analysis (Table 1) not only demonstrated consistent results—MR-Egger (OR, 1.03; 95%CI, 1.01–1.05; *P* = 0.002), MR-PRESSO_corrected_ (OR, 1.03; 95%CI, 1.02–1.04; *P* < 0.001), and MR weighted median (OR, 1.03; 95%CI, 1.02–1.04; *P* < 0.001), but also indicated no directional pleiotropy in MR-Egger test (*P*_intercept_ =0.37). Although the sensitivity analyses using MR-PRESSO suggested potential pleiotropy (*P*_global_ =0.02), the corrected MR-PRESSO yielded an estimate that is consistent with the primary analysis (OR, 1.03; 95%CI, 1.02–1.04; *P* < 0.001) after removing variants with potential pleiotropy.

**Table 1:** Sensitivity analysis using MR Egger, MR-PRESSO, and MR weighted median methods.

	**Inverse variance weighted estimate (primary analysis)**		**MR-Egger**			**MR-PRESSO (raw)**			**MR-PRESSO (corrected)**		**MR weighted median**	
**Outcomes**	**Odds ratio(95% CI)**	***P*-value**	**Odds ratio (95% CI)**	***P*-value**	$P_{\text{intercept}}^{*}$	**Odds ratio (95% CI)**	***P*-value**	***P*_global_^†^**	**Odds ratio (95% CI)**	***P*-value**	**Odds ratio (95% CI)**	***P*-value**
Asthma (overall)	1.02 (1.01–1.03)	0.004	1.03 (1.01–1.05)	0.002	0.37	1.02 (1.01–1.03)	<0.001	0.02	1.03 (1.02–1.04)	<0.001	1.03 (1.02–1.04)	<0.001
Childhood asthma	1.05 (1.02–1.08)	<0.001	1.04 (1.00–1.08)	0.04	0.95	1.04 (1.02–1.07)	<0.001	0.05			1.04 (1.02–1.07)	<0.001
Adult-onset asthma	1.01 (1.00–1.03)	0.01	1.02 (0.99–1.04)	0.058	0.46	1.01 (1.00–1.03)	0.02	0.56			1.02 (1.00–1.03)	0.03
Allergic asthma	1.03 (1.01–1.04)	0.004	1.05 (1.02–1.07)	0.001	0.28	1.03 (1.02–1.05)	<0.001	0.02	1.03 (1.02–1.05)	<0.001	1.04 (1.02–1.06)	<0.001
Non-allergic asthma	1.02 (1.00–1.03)	0.006	1.02 (0.99–1.04)	0.096	0.77	1.02 (1.00–1.03)	0.007	0.63			1.02 (1.00–1.03)	0.04
Obese asthma	1.02(1.01–1.03)	0.007	1.02 (0.99–1.04)	0.26	0.75	1.02 (1.00–1.03)	0.001	0.97			1.02 (0.99–1.04)	0.12
Non-obese asthma	1.02 (1.00–1.04)	0.03	1.04 (1.01–1.06)	0.002	0.25	1.03 (1.01–1.04)	<0.001	0.004	1.03 (1.02–1.04)	<0.001	1.03 (1.02–1.05)	<0.001

*CI, confidence interval*.

*The non-significant P-values of the MR-Egger intercept test (Pintercept) indicate the absence of directional pleiotropy and the absence of violation for the instrument strength independent of direct effect (InSIDE) assumption, and indicate that the inverse variance weighted estimate is unbiased.

† *Significant Pglobal indicates potential horizontal pleiotropy*.

**Figure 2:**
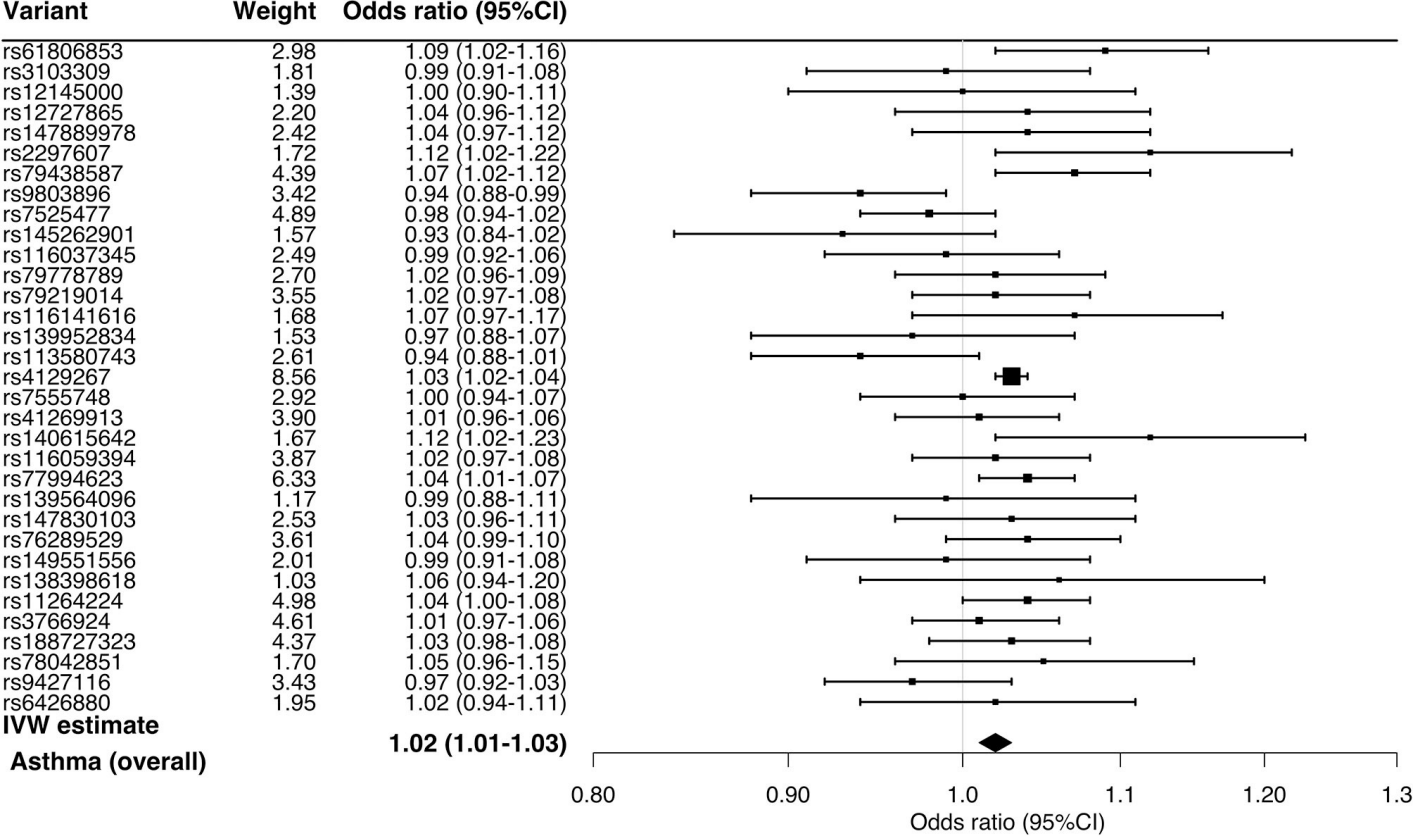
Mendelian randomization estimates for the effect of soluble interleukin-6 receptors on asthma and its phenotypes. By using the inverse variance weighted method, the combined causal effect of sIL-6R on the asthma (overall) outcome and six asthma phenotypes was estimated. The odds ratios were estimated per one standard deviation increment in the inverse-rank normalized sIL-6R level. CI, confidence interval.

In the stratified analysis, the significant association persisted across the asthma phenotypes (Figure 2)—e.g., childhood asthma (OR, 1.05; 95%CI, 1.02–1.08; *P* < 0.001) and obese asthma (OR, 1.02; 95%CI, 1.01–1.03; *P* = 0.007). Likewise, the sensitivity analysis also demonstrated consistent results (Table 1)—e.g., MR-Egger for childhood asthma (OR, 1.04; 95%CI, 1.00–1.08; *P* = 0.04), MR-PRESSO for obese asthma (OR, 1.02; 95%CI, 1.00–1.03; *P* = 0.001), and MR weighted median for childhood asthma (OR, 1.04; 95%CI, 1.02–1.07; *P* < 0.001). Although the sensitivity analyses using MR-PRESSO suggested potential pleiotropy for allergic asthma (*P*_global_ =0.02) and non-obese asthma (*P*_global_ =0.004), the corrected MR-PRESSO yielded an estimate that is consistent with the primary analysis—allergic asthma (OR, 1.03; 95%CI, 1.02–1.05; *P* < 0.001) and non-obese asthma (OR, 1.03; 95%CI, 1.02–1.04; *P* < 0.001)—after removing variants with potential pleiotropy.

**Figure 3:**
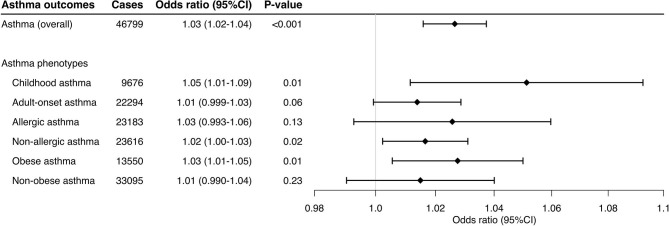
Mendelian randomization estimates for the effect of soluble interleukin-6 receptors on asthma and its phenotypes using different thresholds. This sensitivity analysis used 16 variants selected with the use of more-stringent P_GWAS_ (P_GWAS_ <5 × 10^−8^) and linkage disequilibrium (*r*^2^ < 0.02) thresholds. By using the inverse variance weighted method, the combined causal effect of sIL-6R on the asthma (overall) outcome and six asthma phenotypes was estimated. The odds ratios were estimated per one standard deviation increment in the inverse-rank normalized sIL-6R level. CI, confidence interval.

Lastly, the sensitivity analysis using 16 variants selected by the use of more stringent thresholds—P_GWAS_ <5 × 10^−8^ and linkage disequilibrium (*r*^2^ <0.02)—also demonstrated significant associations with overall asthma (OR, 1.03; 95%CI, 1.02–1.04; *P* <0.001; Supplementary Figure 1) and its phenotypes—e.g., childhood asthma (OR, 1.05; 1.01–1.09; *P* = 0.01) and obese asthma (OR, 1.03; 95%CI, 1.01–1.05; *P* = 0.01; Figure 3).

## Discussion

In this two-sample Mendelian randomization study, we demonstrated that higher genetically-instrumented sIL-6R levels were associated with a modestly but significantly increased risk of overall asthma. The sensitivity analyses also showed consistent results. Our results are in line with recent findings that higher circulating IL-6 levels are associated with a greater exacerbation risk both in children (20) and adults (21, 22). Additionally, genetic studies showed the associations of *IL6R*-related polymorphisms with asthma prevalence and severity, and lower pulmonary function (5, 6). For example, the Severe Asthma Research Program (SARP) cohorts found that rs2228145 is associated with a higher serum sIL-6R level, greater asthma severity, and lower pulmonary function in patients with severe asthma (6). However, their subsequent study also reported discordant results—e.g., no association of sIL-6R level with severity or exacerbation risk (22). The apparent discrepancies between these reports may be attributable to the differences in study design, setting, sample, analytical assumptions, or any combination of these factors. Regardless, the validity of the current study is buttressed by the use of the Mendelian randomization design. This design can mitigate unmeasured confounding and reverse causation that occur with conventional observational studies (13). The current analysis meets the assumptions of Mendelian randomization design in that we identified the genetic variants that are strongly associated with the sIL-6R level (the relevance assumption) and do not share common causes with asthma (the independence assumption), and in that we ensured no effects of known or unknown pleiotropy (the exclusion restriction assumption) (23). The current analysis using the data of two large cohorts corroborates the earlier reports, and extends them by investigating potential causal effects of sIL-6R on asthma and its phenotypes.

The mechanisms underlying our findings remain to be elucidated. For example, inflammatory signals (e.g., C-reactive protein, chemokine ligand 1, IL-1β, IL-8, tumor necrosis factor, bacterial lipopolysaccharides) promote alternative mRNA splicing and shedding of IL-6R from the cell surface, thereby producing sIL-6R and activating the IL-6 trans-signaling pathway (24–29). An analysis of U-BIOPRED data reported that adult patients characterized by IL-6 trans-signaling-related epithelial gene signature had upregulated innate immune pathways, type 2 inflammation-independent eosinophilia, increased submucosal inflammation and airway remodeling, and higher asthma exacerbation rate (25). In patients with obese asthma, their airway inflammation is characterized by dominance of neutrophils and macrophages—major sources of sIL-6R in both lungs (5) and adipose tissue (30). Experimental asthma models also demonstrated that IL-6R inhibitors attenuate the airway inflammatory response characterized by mixed granulocytic infiltration with elevated IL-6 and IL-6R levels (31). These data collectively present a rationale for targeting the IL-6 trans-signaling pathway in asthma. We acknowledge that, in the current analysis, the observed magnitude of estimates was nominally small. Nevertheless, our findings encourage further investigations into identifying patients with specific asthma endotype(s) in which IL-6 pathways play major roles [e.g., patients with adiposopathy—“sick fat” (30)].

This study has potential limitations. First, misclassification of asthma and its phenotypes is possible, while it is unrelated to the measured sIL-6R levels in the current two-sample design. Therefore, this independent non-differential misclassification would have biased the inferences toward the null. Second, the small sample size of patients with moderate-to-severe asthma precluded us from robustly examining this specific group that may benefit more from anti-IL-6 therapies. Third, as with any Mendelian randomization study, survivor (selection) bias is possible. However, participants from the INTERVAL study (mean age, 44 years) and UK Biobank (mean age, 57 years) were not in age ranges where survivor bias imposes a substantial impact. Fourth, the current study design using summary statistics precluded us from evaluating a potential non-linear relationship of sIL-6R with the asthma outcomes. Fourth, the influence of genetic instruments may be abated or buffered by feedback mechanisms or developmental processes. Yet, such mechanisms would have diminished the genetic effects, thereby biasing the inferences toward the null. Lastly, to minimize the population stratification bias, we restricted the study sample to individuals of European ancestry. Therefore, the inferences may not be generalizable to other racial/ethnic populations.

In conclusion, the current Mendelian randomization study using two large cohort data demonstrated that higher genetically-instrumented sIL-6R levels are associated with a significantly but modestly increased risk of overall asthma. The observation was consistent across the asthma phenotypes and different assumptions. Our inferences support further research into delineating the roles of IL-6 pathways in the asthma pathobiology and identifying patients with a distinct endotype who would benefit most from anti-IL-6 therapies.

## Data Availability Statement

The raw data supporting the conclusions of this article will be made available by the authors, without undue reservation.

## Ethics Statement

The studies involving human participants were reviewed and approved by all participants from this study provided UK Biobank-acquired informed consent and provided data according to the UK Biobank protocol. The current study has complied with all ethical regulations according to UK Biobank policy. This research was approved and conducted using the UK Biobank under application number 16549 and 45052. The institutional review board of Harvard University and Massachusetts General Hospital approved the study. The patients/participants provided their written informed consent to participate in this study.

## Author Contributions

YR carried out the main statistical analysis, drafted the initial manuscript, and approved the final manuscript as submitted. ZZ created the summary statistics of UK Biobank, drafted the initial manuscript, and approved the final manuscript as submitted. CC conceptualized and designed the study, supervised the conduct of study, critically reviewed and revised the initial manuscript, and approved the final manuscript as submitted. RF assisted study design, reviewed the manuscript, and approved the final manuscript. LL conceptualized the study, obtained funding and reviewed the manuscript, and approved the final manuscript. DN conceptualized the study, reviewed the manuscript, and approved the final manuscript. KH conceptualized the study, obtained funding, supervised the statistical analysis, reviewed and revised the initial manuscript, and approved the final manuscript as submitted. All authors contributed to the article and approved the submitted version.

## Conflict of Interest

CC has participated in scientific advisory boards for AstraZeneca and GSK. KH has received a research grant from Novartis. The remaining authors declare that the research was conducted in the absence of any commercial or financial relationships that could be construed as a potential conflict of interest.
