# Supplementary material for: Relationship of Soluble Interleukin-6 Receptors With Asthma: A Mendelian Randomization Study
Source: Front Med (Lausanne). 2021 Apr 12;8:665057. doi: 10.3389/fmed.2021.665057 (PMC8071981; doi:10.3389/fmed.2021.665057)
Supplement: Supplementary file 1 [file Data_Sheet_1.docx]

Supplementary Material

**Relationship of soluble interleukin-6 receptors with asthma: A Mendelian randomization study**

**Authors**: Yoshihiko Raita, MD, MPH, MMSc; Zhaozhong Zhu, ScD; Carlos A. Camargo, Jr., MD, DrPH; Robert J. Freishtat, MD, MPH; Debby Ngo, MD; Liming Liang, PhD; and Kohei Hasegawa, MD, MPH

**CONTENTS**

**Supplementary Methods** …………………..………………………………..…………………...…...…………2

**Supplementary References**……………….…………………………………………………......…………..…..4

**Supplementary Table 1.** Characteristics of the selected 32 variants strongly associated with soluble interleukin-6 receptors level (P_GWAS_$<$5$\times$10^-6^, $r^{2}<$0.1)………………………………….……………………….6

**Supplementary Figure 1.** Mendelian randomization estimates for the effect of soluble interleukin-6 receptors on overall asthma outcome using different thresholds……………………………………………………………8

**METHODS**

**Data Summary, Quality Control, and Imputation of Genotyping Data**

*The INTERVAL study*

Detailed information of the INTERVAL study was described elsewhere (1). In brief, approximately 50,000 blood donors aged $\geq$18 years were recruited at 25 centers of England’s National Health Service Blood and Transplant from 2012 to 2014. For proteomic profiling, randomly selected two non-overlapping subcohorts of 2,731 and 831 participants of European ancestry were enrolled. The levels of 2,994 plasma proteins were measured by the use of SomaLogic assays, a validated high-throughput and aptamer-based method (2–4). Protein levels with relative fluorescent unit were transformed using natural log transformation and adjusted in a linear regression for age, sex, duration between blood draw and processing (binary, ≤1 vs. >1 day), and the first three principal components of ancestry from multi-dimensional scaling. The protein residuals from this linear regression model were then rank-inverse normalized, and used as the phenotypes for the genome-wide association analysis. After quality control (QC) for genotyping, 3,301 participants (2,481 and 820 in the two subcohorts) remained for the analysis. Linear regression using an additive genetic model was used to test genetic associations. Association tests were conducted on allelic dosages to account for imputation uncertainty (‘-method expected’ option) using SNPTEST v2.5.2 for 10.6 million imputed autosomal variants (5). The results of two subcohorts of the study were combined using fixed-effect inverse variance meta-analysis. Overall, 1,927 significant (P_GWAS_$<$1.5×10^−11^) associations between 764 genomic regions and 1,478 proteins were identified. The summary statistics data are publicly available at <http://www.phpc.cam.ac.uk/ceu/proteins/>.

*The UK Biobank*

The details of genotyping procedures in the UK Biobank were described on the UK Biobank website (http://biobank.ctsu.ox.ac.uk/) and in previous research (6,7). In summary, approximately 500,000 participants aged 40-69 years were enrolled in 2006-2010. The study has collected extensive phenotypic (e.g., demographics, medical history, anthropomorphic measures) and genotypic data. After QC, the current UK Biobank data contain approximately 800,000 markers that passed SNP QC in at least one batch. The genotype imputation was performed using the UK10k+1000 Genome Phase 3 panels; genotype imputation procedure provided over 90 million variants (8,9). The current study restricted the sample to subjects of European ancestry to minimize population stratification. All participants from this study provided UK Biobank-acquired informed consent and provided data according to the UK Biobank protocol. The current study has complied with all ethical regulations according to UK Biobank policy. This research was approved and conducted using the UK Biobank under application number 16549 and 45052.

**Ascertainment of Asthma and its Phenotypes**

First, according to previous research (7,10,11), we identified all UK Biobank participants with asthma by using the data fields 6152 (self-reported physician-diagnosis of asthma), 20002 (non-cancer illness disease code), 41202 (*International Classification of Diseases, Tenth Revision, Clinical Modification* [*ICD-10-CM*] primary diagnosis in the hospital), and 41204 (*ICD-10-CM* secondary diagnosis in the hospital), with the use of the *ICD-10-CM* diagnosis codes of J45. We excluded individuals with chronic obstructive pulmonary disease, emphysema or chronic bronchitis (self-reports or International Statistical Classification of Diseases and Related Health Problems, 10th Revision codes) from cases with asthma and controls. In the current study, the primary outcome was (overall) asthma (n=46,799) and the secondary outcomes were six major asthma phenotypes: 1) childhood asthma (defined as age of onset $\leq$12 years; n=9,676)(11), 2) adult-onset asthma (defined as age of onset $\geq$26 years; n=22,294)(11), 3) allergic asthma (defined as asthma with an allergic disease—eczema, food allergy, and/or allergy rhinitis [identified by data fields 6152, 20002, 41202, 41204]; n=23,183)(7,10), 4) non-allergic asthma (defined as asthma without any allergic disease; n=23,616), 5) obese asthma (defined as BMI of$\geq$30 kg/m^2^; n=13,550), and 6) non-obese asthma (defined as BMI of $<$30 kg/m^2^; n=33,095). Young adult-onset asthma (age of onset 12.1–25 years) was not included in the genetic analysis due to its higher heterogeneity (11–13). We also identified shared controls (n=347,457) with high-quality genotyping and complete phenotype/covariate data for GWAS association analysis. To account for relatedness, the association test between imputed variants and asthma traits in UK Biobank data and was carried out using BOLT-linear mixed model (LMM). The output of BOLT-LMM linear regression was transformed into log odds ratio (log odds ratio) for HBP binary phenotype (11,14).

**Statistical Analysis**

As genetic instruments, we first selected 40 independent *cis*-acting variants strongly associated with soluble interleukin-6 receptor (sIL-6R) levels (the relevance condition) (P_GWAS_$<$5$\times$10^-6^,$r$^2^<0.1, 250kb from *IL6R*) in the INTERVAL study. One variant (rs5777914) is missing from the outcome GWAS summary statistics. We searched LD proxy ($r$^2^> 0.8) using *LDlinkR* package(15), but LD proxy for this variant was not identified. We then removed six palindromic variants because of the lack of availability of minor allele frequency data in the INTERVAL study, leaving 33 independent *cis*-acting variants for the current analysis (**Supplementary Table** **1**). To ensure no confounding for variants (the independence condition), we investigated their Bonferroni-corrected associations with potential confounders—education, smoking, and physical activity—using publicly available summary statistics in the UK Biobank (16). We also assured that variants affect outcomes solely through sIL-6R (the exclusion restriction condition) by examining the variants in three curated genetic cross-reference systems—Ensembl (17), GWAS catalog (18), and PhenoScanner (19). To investigate the association of variants with outcomes, we used GWAS summary statistics of the UK Biobank. We weighted the magnitude of association of each variant with outcomes by that with sIL-6R, and combined causal estimates of sIL-6R on each outcome by the inverse-variance weighted meta-analysis method with a random-effects model (20,21) using *MendelianRandomization* package (22).

In the sensitivity analyses, we first applied MR-Egger regression (23), MR Pleiotropy RESidual Sum and Outlier (MR-PRESSO) test (24), and MR weighted median method (25). MR-Egger regression detects pleiotropy based on the assumption that the pleiotropic associations are independent from the genetic associations with the exposure (i.e., the instrument strength independent of direct effect [InSIDE] assumption) and provides corrected estimates. MR-PRESSO test(24) detects violation of the restriction exclusion criterion assumption and provides corrected estimates by removing variants which exhibit pleiotropy. MR weighted median method provides consistent estimates even when 50% of the information comes from invalid variants. We conducted MR-Egger regression and weighted median method using *MendelianRandomization* package(22) and MR-PRESSO using *MRPRESSO* package (24). Second, we also used more-stringent P_GWAS_ (P_GWAS_$<$5$\times$10^-8^) and linkage disequilibrium ($r^{2}<$0.02) thresholds to examine the robustness of the inferences. We analyzed the data using R version 3.6.3 (R foundation for Statistical Computing, Vienna, Austria).

**SUPPLEMENTARY REFERENCES**

1. Sun BB, Maranville JC, Peters JE, Stacey D, Staley JR, Blackshaw J, Burgess S, Jiang T, Paige E, Surendran P, et al. Genomic atlas of the human plasma proteome. *Nature* (2018) **558**:73–79. doi:10.1038/s41586-018-0175-2

2. Ngo D, Sinha S, Shen D, Kuhn EW, Keyes MJ, Shi X, Benson MD, O’Sullivan JF, Keshishian H, Farrell LA, et al. Aptamer-based proteomic profiling reveals novel candidate biomarkers and pathways in cardiovascular disease. *Circulation* (2016) **134**:270–285. doi:10.1161/CIRCULATIONAHA.116.021803

3. Kraemer S, Vaught JD, Bock C, Gold L, Katilius E, Keeney TR, Kim N, Saccomano NA, Wilcox SK, Zichi D, et al. From SOMAmer-based biomarker discovery to diagnostic and clinical applications: a SOMAmer-based, streamlined multiplex proteomic assay. *PLoS ONE* (2011) **6**:e26332. doi:10.1371/journal.pone.0026332

4. Hensley P. SOMAmers and SOMAscan – A protein biomarker discovery platform for rapid analysis of sample collections from bench top to the clinic. *J Biomol Tech* (2013) **24**:S5.

5. Marchini J, Howie B, Myers S, McVean G, Donnelly P. A new multipoint method for genome-wide association studies by imputation of genotypes. *Nat Genet* (2007) **39**:906–913. doi:10.1038/ng2088

6. Sudlow C, Gallacher J, Allen N, Beral V, Burton P, Danesh J, Downey P, Elliott P, Green J, Landray M, et al. UK biobank: an open access resource for identifying the causes of a wide range of complex diseases of middle and old age. *PLoS Med* (2015) **12**:e1001779. doi:10.1371/journal.pmed.1001779

7. Zhu Z, Lee PH, Chaffin MD, Chung W, Loh P-R, Lu Q, Christiani DC, Liang L. A genome-wide cross-trait analysis from UK Biobank highlights the shared genetic architecture of asthma and allergic diseases. *Nat Genet* (2018) **50**:857–864. doi:10.1038/s41588-018-0121-0

8. 1000 Genomes Project Consortium, Auton A, Brooks LD, Durbin RM, Garrison EP, Kang HM, Korbel JO, Marchini JL, McCarthy S, McVean GA, et al. A global reference for human genetic variation. *Nature* (2015) **526**:68–74. doi:10.1038/nature15393

9. UK10K Consortium, Walter K, Min JL, Huang J, Crooks L, Memari Y, McCarthy S, Perry JRB, Xu C, Futema M, et al. The UK10K project identifies rare variants in health and disease. *Nature* (2015) **526**:82–90. doi:10.1038/nature14962

10. Zhu Z, Guo Y, Shi H, Liu C-L, Panganiban RA, Chung W, O’Connor LJ, Himes BE, Gazal S, Hasegawa K, et al. Shared genetic and experimental links between obesity-related traits and asthma subtypes in UK Biobank. *J Allergy Clin Immunol* (2020) **145**:537–549. doi:10.1016/j.jaci.2019.09.035

11. Zhu Z, Zhu X, Liu C-L, Shi H, Shen S, Yang Y, Hasegawa K, Camargo CA, Liang L. Shared genetics of asthma and mental health disorders: a large-scale genome-wide cross-trait analysis. *Eur Respir J* (2019) **54**: doi:10.1183/13993003.01507-2019

12. Pividori M, Schoettler N, Nicolae DL, Ober C, Im HK. Shared and distinct genetic risk factors for childhood-onset and adult-onset asthma: genome-wide and transcriptome-wide studies. *Lancet Respir Med* (2019) **7**:509–522. doi:10.1016/S2213-2600(19)30055-4

13. Ferreira MAR, Mathur R, Vonk JM, Szwajda A, Brumpton B, Granell R, Brew BK, Ullemar V, Lu Y, Jiang Y, et al. Genetic architectures of childhood- and adult-onset asthma are partly distinct. *Am J Hum Genet* (2019) **104**:665–684. doi:10.1016/j.ajhg.2019.02.022

14. Loh P-R, Tucker G, Bulik-Sullivan BK, Vilhjálmsson BJ, Finucane HK, Salem RM, Chasman DI, Ridker PM, Neale BM, Berger B, et al. Efficient Bayesian mixed-model analysis increases association power in large cohorts. *Nat Genet* (2015) **47**:284–290. doi:10.1038/ng.3190

15. CRAN - Package LDlinkR. Available at: https://cran.r-project.org/web/packages/LDlinkR/index.html [Accessed February 5, 2021]

16. UK Biobank. *Neale lab* Available at: http://www.nealelab.is/uk-biobank [Accessed February 5, 2021]

17. Ensembl genome browser 101. Available at: https://uswest.ensembl.org/index.html [Accessed November 2, 2020]

18. GWAS Catalog. Available at: https://www.ebi.ac.uk/gwas/ [Accessed November 2, 2020]

19. PhenoScanner. Available at: http://www.phenoscanner.medschl.cam.ac.uk/ [Accessed November 2, 2020]

20. Bowden J, Spiller W, Del Greco M F, Sheehan N, Thompson J, Minelli C, Davey Smith G. Improving the visualization, interpretation and analysis of two-sample summary data Mendelian randomization via the Radial plot and Radial regression. *Int J Epidemiol* (2018) **47**:1264–1278. doi:10.1093/ije/dyy101

21. Burgess S, Dudbridge F, Thompson SG. Combining information on multiple instrumental variables in Mendelian randomization: comparison of allele score and summarized data methods. *Stat Med* (2016) **35**:1880–1906. doi:10.1002/sim.6835

22. Staley OYJ. *MendelianRandomization: Mendelian Randomization Package*. (2020). Available at: https://CRAN.R-project.org/package=MendelianRandomization [Accessed November 2, 2020]

23. Burgess S, Thompson SG. Interpreting findings from Mendelian randomization using the MR-Egger method. *Eur J Epidemiol* (2017) **32**:377–389. doi:10.1007/s10654-017-0255-x

24. Verbanck M, Chen C-Y, Neale B, Do R. Detection of widespread horizontal pleiotropy in causal relationships inferred from Mendelian randomization between complex traits and diseases. *Nature Genetics* (2018) **50**:693–698. doi:10.1038/s41588-018-0099-7

25. Bowden J, Davey Smith G, Haycock PC, Burgess S. Consistent estimation in Mendelian randomization with some invalid instruments using a weighted median estimator. *Genet Epidemiol* (2016) **40**:304–314. doi:10.1002/gepi.21965

**Supplementary Table 1. Characteristics of the selected 33 variants strongly associated with soluble interleukin-6 receptors level (P_GWAS_**$\boldsymbol{<}$**5**$\boldsymbol{\times}$**10^-6^,** $\boldsymbol{r}^{\boldsymbol{2}}\boldsymbol{<}$**0.1)**

| Variant | Consequence | Effect allele | Association with sIL-6R | | *F*-statistic | Association with three potential confounders in the UK Biobank (Bonferroni-corrected P-value) | | |
| --- | --- | --- | --- | --- | --- | --- | --- | --- |
|  |  |  | coefficient | log_10_(P-value) |  | Education | Smoking status | Physical activity |
| rs61806853 | non_coding | T | -0.50 | -17.3 | 74.84 | 0.99 | 0.99 | 0.99 |
| rs3103309 | non_coding | C | -0.16 | -9.07 | 37.58 | 0.99 | 0.99 | 0.99 |
| rs12145000 | non_coding | C | -0.48 | -7.68 | 31.36 | 0.99 | 0.99 | 0.99 |
| rs12727865 | non_coding | C | 0.19 | -12.05 | 51.08 | 0.99 | 0.99 | 0.99 |
| rs147889978 | non_coding | G | 0.56 | -12.73 | 54.17 | 0.99 | 0.76 | 0.99 |
| rs2297607 | synonymous | A | 0.18 | -8.78 | 36.41 | 0.99 | 0.99 | 0.99 |
| rs79438587 | non_coding | C | -0.41 | -32.39 | 143.57 | 0.51 | 0.99 | 0.99 |
| rs9803896 | non_coding | G | -0.33 | -22.46 | 98.40 | 0.99 | 0.99 | 0.99 |
| rs7525477 | non_coding | G | 0.35 | -40.32 | 180.03 | 0.99 | 0.99 | 0.99 |
| rs145262901 | non_coding | G | -0.72 | -11.01 | 46.33 | NA | NA | NA |
| rs116037345 | non_coding | C | 0.58 | -15.15 | 65.17 | 0.99 | 0.99 | 0.99 |
| rs79778789 | non_coding | A | -0.79 | -18.08 | 78.36 | 0.99 | 0.99 | 0.99 |
| rs79219014 | non_coding | G | -0.76 | -22.34 | 97.72 | 0.99 | 0.99 | 0.99 |
| rs116141616 | non_coding | G | 0.45 | -9.34 | 38.81 | 0.99 | 0.99 | 0.99 |
| rs139952834 | non_coding | C | 0.65 | -8.89 | 36.83 | 0.99 | 0.99 | 0.99 |
| rs113580743 | non_coding | G | 0.51 | -16.71 | 72.21 | 0.99 | 0.99 | 0.99 |
| rs4129267 | non_coding | C | -1.11 | -1100.13 | 5041.90 | 0.99 | 0.99 | 0.99 |
| rs7555748 | non_coding | A | -0.53 | -17.01 | 73.60 | 0.99 | 0.99 | 0.99 |
| rs41269913 | non_coding | C | -0.70 | -30.71 | 135.90 | 0.99 | 0.99 | 0.99 |
| rs140615642 | non_coding | T | -0.55 | -10.2 | 42.78 | 0.99 | 0.99 | 0.99 |
| rs116059394 | non_coding | A | 0.58 | -24.06 | 105.76 | 0.99 | 0.99 | 0.99 |
| rs77994623 | non_coding | C | 0.62 | -89.56 | 405.75 | 0.99 | 0.99 | 0.99 |
| rs139564096 | non_coding | G | 0.64 | -5.55 | 21.93 | 0.99 | 0.99 | 0.99 |
| rs147830103 | non_coding | G | 0.65 | -13.86 | 59.27 | 0.99 | 0.99 | 0.99 |
| rs76289529 | non_coding | C | -0.67 | -22.01 | 96.38 | 0.99 | 0.99 | 0.99 |
| rs149551556 | non_coding | C | 0.67 | -11.66 | 49.33 | 0.99 | 0.99 | 0.99 |
| rs138398618 | non_coding | G | 0.50 | -6.04 | 24.12 | 0.99 | 0.99 | 0.99 |
| rs11264224 | non_coding | A | -0.46 | -41.41 | 185.48 | 0.99 | 0.99 | 0.99 |
| rs3766924 | non_coding | C | 0.39 | -37.1 | 165.81 | 0.99 | 0.18 | 0.99 |
| rs188727323 | non_coding | C | -0.41 | -34.98 | 155.46 | 0.99 | 0.99 | 0.99 |
| rs78042851 | non_coding | A | -0.45 | -9.09 | 37.69 | 0.99 | 0.99 | 0.99 |
| rs9427116 | non_coding | T | -0.24 | -22.22 | 97.15 | 0.99 | 0.99 | 0.99 |
| rs6426880 | non_coding | G | 0.26 | -9.36 | 38.96 | 0.99 | 0.99 | 0.99 |
| Results of rs145262901 were not identified in the publicly available summary styatistics. | | | | | | | |  |

**Supplementary Figure 1. Mendelian randomization estimates for the effect of soluble interleukin-6 receptors on overall asthma outcome using different thresholds**

This sensitivity analysis used 16 variants selected with the use of more-stringent P_GWAS_ (P_GWAS_<5×10^-8^) and linkage disequilibrium (r^2^<0.02) thresholds. The size of the squares is proportional to the weight of the Mendelian randomization estimate for each variant, with the horizontal lines indicating their 95% confidence intervals. The center of the diamond represents the combined Mendelian randomization point estimate with the lateral tips indicating its 95% confidence interval, estimated by the inverse variance weighted method. The odds ratios were estimated per one standard deviation increment in the inverse-rank normalized sIL-6R level.

Abbreviations: CI, confidence interval; IVW, inverse variance weighted

**
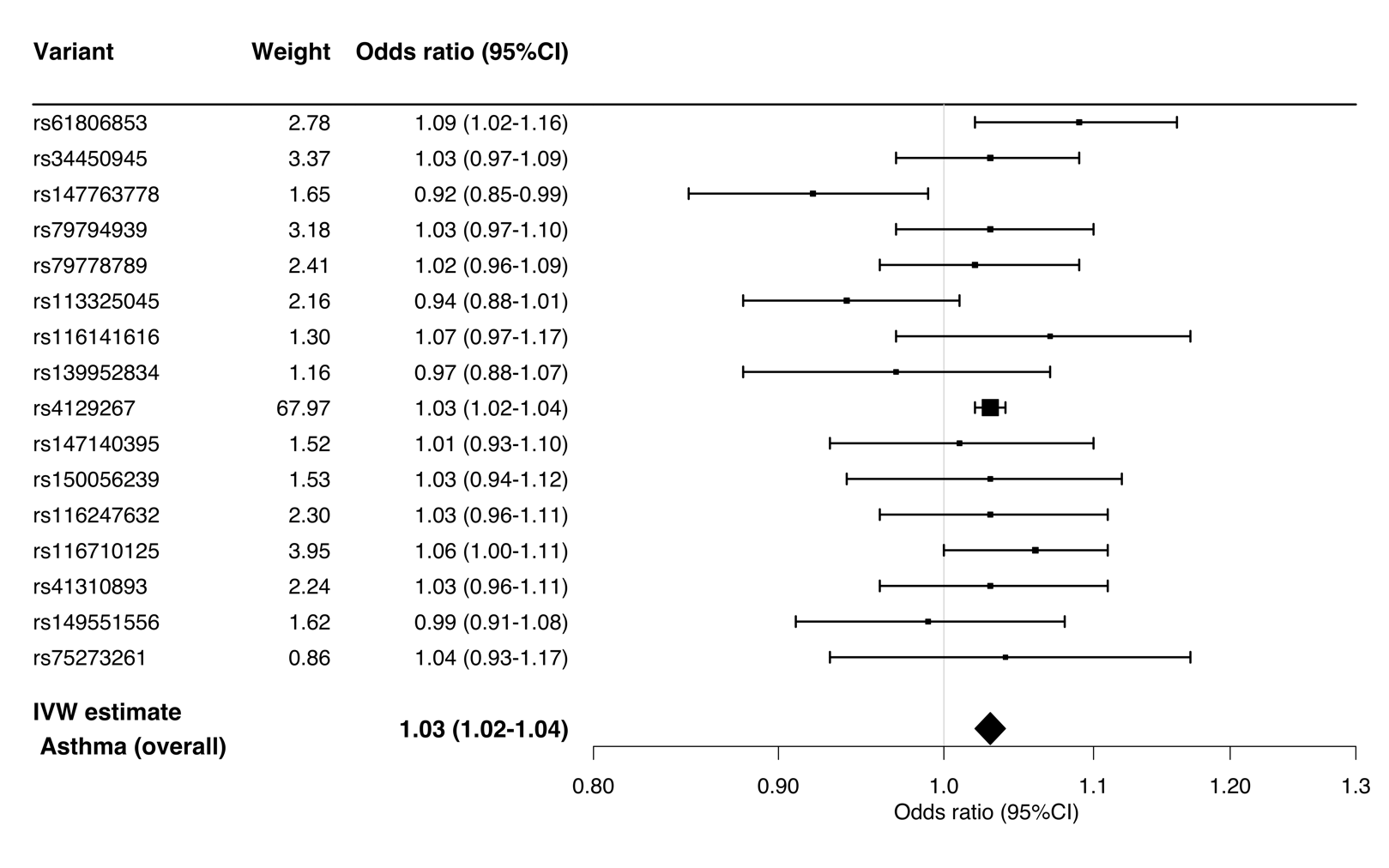
**
